# Current State of Dendritic Cell-Based Immunotherapy: Opportunities for *in vitro* Antigen Loading of Different DC Subsets?

**DOI:** 10.3389/fimmu.2018.02804

**Published:** 2018-12-03

**Authors:** Anne Huber, Floris Dammeijer, Joachim G. J. V. Aerts, Heleen Vroman

**Affiliations:** ^1^Department of Pulmonary Medicine, Erasmus Medical Center, Rotterdam, Netherlands; ^2^Erasmus Cancer Institute, Erasmus Medical Center, Rotterdam, Netherlands

**Keywords:** dendritic cell (DC), Immunotherapy, DC subsets, T cell responses, tumor immunology

## Abstract

Dendritic cell (DC) based cancer immunotherapy aims at the activation of the immune system, and in particular tumor-specific cytotoxic T lymphocytes (CTLs) to eradicate the tumor. DCs represent a heterogeneous cell population, including conventional DCs (cDCs), consisting of cDC1s, cDC2s, plasmacytoid DCs (pDCs), and monocyte-derived DCs (moDCs). These DC subsets differ both in ontogeny and functional properties, such as the capacity to induce CD4^+^ and CD8^+^ T-cell activation. MoDCs are most frequently used for vaccination purposes, based on technical aspects such as availability and *in vitro* expansion. However, whether moDCs are superior over other DC subsets in inducing anti-tumor immune responses, is unknown, and likely depends on tumor type and composition of the tumor microenvironment. In this review, we discuss cellular aspects essential for DC vaccination efficacy, and the most recent findings on different DC subsets that could be used for DC-based cancer immunotherapy. This can prove valuable for the future design of more effective DC vaccines by choosing different DC subsets, and sheds light on the working mechanism of DC immunotherapy.

## Introduction

The immune system is able to distinguish between self, non-self and eliminate damaged cells. Consequently, it has the potential to eradicate cancerous cells displaying mutated, or aberrantly expressed self-antigens. To avoid elimination by immune responses, tumors not only acquire the ability to prevent immune recognition, but also create an immunosuppressive environment and actively hijack immune cells to aid in tumor progression (1, 2). Re-activating the immune system to treat patients with cancer was already proposed at the end of the nineteenth century and cancer immunotherapy has further developed ever since (3–6). One type of immunotherapy is dendritic cell (DC) vaccination (7). DC vaccination makes use of autologous DCs loaded *ex-vivo* with specific tumor-associated antigens (TAAs) or whole tumor lysate to generate an immune response aiming for cancer-cell elimination. DC vaccination using *ex-vivo* generated monocyte-derived DCs (moDCs) in patients with cancer was first explored over two decades ago (8). Numerous clinical trials [over 200 (9)] have established the safety and ability of moDC vaccines to induce anti-tumor responses (10–12). More recently, also *in vivo* loading of DCs is being exploited (13–17). In this review, we will discuss the cellular aspects essential for DC vaccination efficacy, the potential of distinct DC subsets as sources for DC vaccination, and the implications for the future design of DC vaccines.

## Dendritic Cells

DCs play a crucial role in the immune system and link innate and adaptive immune responses (18–21). They arise from progenitor cells in the bone marrow and reside in peripheral tissues in an immature state. Immature DCs (iDCs) are specialized in antigen capturing, processing, and presentation. Upon appropriate stimulation mediated by inflammatory and pathogen-derived signals, iDCs undergo maturation. Mature DCs express co-stimulatory molecules, secrete cytokines, and migrate to lymphoid organs where they activate antigen-specific T-cells (22). Besides the presentation of exogenous antigens on MHC-II peptides, DCs are able to cross-present exogenously captured antigens on MHC I-associated peptides (23). Thereby, DCs can present TAAs to CD8^+^ T-cells which makes them of particular interest for cancer immunotherapy (24).

DCs consist of developmentally and functionally distinct DC subsets. These include moDCs, conventional DCs—consisting of cDC1s and cDC2s—and plasmacytoid DCs (pDCs) (25–27). While moDCs are derived from the common monocyte progenitors (cMoPs), cDCs, and pDCs arise from a common DC precursor (27–29). Each DC subset has specialized functions however, these are not exclusive and seem to depend on both location and environmental cues (30). In general, moDCs efficiently promote T-cell differentiation, but are poor inducers of CD4^+^ T-cell proliferation (31). In contrast, moDCs can be powerful activators of tumor-specific CD8^+^ T-cells (32). It is known that mature moDCs secrete chemokines and pro-inflammatory cytokines which are crucial to attract other immune cells and T-cells to the local environment (33). cDC1s are specialized in recognizing viral and intracellular antigens and are important for cytotoxic T-cell (CTL) responses, whereas cDC2s are particularly apt in priming CD4^+^ T-cells (34). Depending on the experimental model, cDC2s induce T-helper (Th) 2 or Th17 responses (35, 36). pDCs are prominent producers of type I interferon in response to single-stranded RNA and double-stranded DNA upon e.g., viral infections, which is important for DC maturation and CD8^+^ T-cell activation (34, 37). However, their antigen-presenting capacity is being questioned, especially as it was recently discovered that pDC characterized by CD123 expression and BDCA2 are contaminated by pre-cDCs (38, 39).

## DC Vaccines

DC-based cancer immunotherapy depends on the crucial role that DCs play in inducing antigen-specific T-cell responses (40). In many tumors, immune responses are ineffective due to the immunosuppressive environment of the tumor and/or the lack of immunogenicity of the tumor (41, 42). In addition, the tumor microenvironment (TME) promotes exhaustion of effector CD8^+^ T-cells (43). Some tumors are even able to hamper the recruitment of cDC1s, by downregulating CCL4 signaling upon constitutively active β-catenin signaling and thereby hamper priming and accumulation of tumor-infiltrating T-cells (44), indicating the importance of endogenous DCs for initiating anti-tumor immunity. DC vaccines aim to overcome the absence or malfunctioning of endogenous DCs by manipulating autologous DCs to enhance T-cell responses directed against the tumor.

Currently a wide range of procedures to generate autologous DCs exist using distinct sources, such as peripheral blood monocytes, naturally occuring DCs, or CD34^+^ hematopoietic precursor cells mobilized from the bone marrow (10), enabling the generations of various DC subsets [such as moDCs, cDCs, or pDCs (45–47)]. In addition, different sources of TAAs [e.g., cancer cell line lysate, whole tumor lysate, or tumor-associated peptides (45, 48, 49)], as well as different antigen-loading methods [such as pulsing, via viral vectors, or mRNA transfection (10)] are used to load DCs. Moreover, various maturation methods including cytokines, CD40 ligands, and TLR agonists (50) are known. Currently, there is a great effort made in improving existing DC vaccines and developing new ones. New approaches include genetically engineered DCs that express TAAs or display enhanced immunostimulatory properties or explore *in vivo* antigen loading of DCs with freshly released TAAs due to chemotherapy or immunogenic tumor-cell death (51–58).

## Generation of Patient-Derived DCs *ex vivo*

Because DCs comprise < 1% of peripheral blood mononuclear cells (PBMCs), one major challenge is the generation of sufficient numbers of DCs for vaccination purposes. Therefore, DC vaccination studies frequently used moDCs that can be generated *ex-vivo* in large numbers from purified monocytes that were consequently cultured with granulocyte-macrophage colony-stimulating factor (GM-CSF) and interleukin (IL)-4 (59). Recently, it was described that monocytes cultured with GM-CSF and IL-6, and activated with IFN-γ, give rise to a newly described mo-cDC1s population that has similarities to cDC1s (60). In addition, cDCs and pDCs can be generated from CD34^+^ hematopoietic stem cells using fms-like tyrosine kinase 3 ligand (Flt3L) (61, 62).

The phenotype, function and ability to induce T-cell responses by *in vitro* generated DCs is highly dependent on the culture methods used (63). For instance, culturing human monocytes with CD137 protein generates DCs potent in inducing CD8^+^ T-cells with superior lysing capabilities against cells infected with cancer-causing viruses (64, 65). Comparing different technologies for monocyte isolation demonstrated that isolation techniques can also influence the antitumor immunogenicity and cytokine production of the generated moDCs (66, 67). Furthermore, the cytokines and growth factors required for precursor-cell differentiation into DCs and subsequent activation influence DC function, and in consequence, the effectivity of DC vaccines (68–71).

## Lymph Node Homing of Vaccinated DCs

To activate antigen-specific T-cell responses, DCs need to reach the lymph nodes (LNs) in order to present antigen to cognate TAA-specific T-cells. In order to optimize DC-trafficking to the LN, various injection routes and strategies have been explored. In a pre-clinical mouse study, different vaccination routes were compared to load DCs *in vivo* with naked antigen-encoded RNA. Herein it was shown that only intra-nodal (i.n) vaccination induced potent expansion of antigen-specific T-cells resulting in prolonged survival, which was not observed upon intra-dermal (i.d.), subcutaneous, or near nodal vaccination (72), indicating the superiority of i.n. vaccination. However, in various clinical studies superior efficacy of i.n. vaccination was less clear. In one study, moDCs pulsed with three melanoma peptides were administered either i.d. or i.n. to 25 patients with metastatic melanoma. After i.d. administration, 4% of DCs migrated to the LNs, whereas migration upon i.n. injection varied between 0 and 56%. The total number of vaccinated moDCs in single LNs were 10- to 30-fold higher after i.n. administration than i.d. injection. However, surprisingly, there was no difference in the strength of the immune response evaluated by TAA-specific CD8^+^ T-cells isolated from DTH reactions between the two administration routes (73). Another study in 54 patients with different types of HER2^+^ breast cancer employed moDCs loaded with six HER2 MHC class II binding peptides injected intralesionally, i.n. or both. More than 80% of the patients had new or increased systemic anti-HER2 CD4^+^ or CD8^+^ T-cell responses and 32 patients had a HER2-specific CD4^+^ T-cell response in the sentinel LN (SLN) after vaccination but these were not significantly different between the three administration routes (74). The large variation observed upon i.n. vaccination also stress the difficulty of i.n. vaccination over i.d. vaccination, and could indicate that accurate i.n. vaccination outperforms i.d. vaccination. It has also been shown that migration to the LNs upon i.d. vaccination can be improved by pre-treating the vaccination site with a potent recall antigen, as tetanus/diphtheria (Td) toxoid pretreatment. This improved DC migration to the LNs, progression free survival and overall survival in patients with glioblastoma (75). Strikingly, systemic TAA-specific immune responses and enhanced tumor CD8^+^ T-cell infiltration were even observed upon intra-tumoral injection of DCs containing an vector expressing the *CCL21* gene in 16 patients with advanced non-small cell lung carcinoma (NSCLC) (54).

Therefore, the superior route or site of injection is still unknown, as no differences were found in safety or antigen-specific immune responses upon either intradermal or -nodal injection (73, 74). These results further urge the need to compare DC vaccination efficacy between different administration routes.

## Evaluation of Efficacy of *ex vivo* Generated moDC Vaccines

As the molecular underpinnings of an effective DC-therapy induced T-cell response are still incompletely understood, it has been difficult to identify factors associated with therapeutic success. As the location and mechanism of T-cell immune responses initiated upon DC therapy is unknown, there is also no consensus how DC vaccination efficacy should be evaluated. One effort to generalize the monitoring of effectivity is by the Response Evaluation Criteria in Solid Tumors (RECIST) or by the more recently described modified RECIST, which enables categorization of patient responses into complete response, partial response, stable disease and progressive disease determined by the amount of tumor shrinkage of a given number of tumor lesions, disease progression, and assessment of pathological LNs (76, 77). Nevertheless, various studies monitored response differently and focused on either clinical responses (summarized in Table 1) or different aspects of the immune response. Moreover, most studies failed to find significant correlations of measured immune characteristics and clinical outcome.

**Table 1:** Clinical trials employing different DC subsets and different sources of antigens.

**DC subset**	**Loading with**	**No. of patients**	**Tumor type**	**Vaccination procedure**	**Clinical outcome**	**References**
moDC	Autologous lysate	10	Epithelial MPM	Three vaccinations i.d. (1/3) and i.v. (2/3) in at 0, 2 and 4 weeks	CT scans and chest X-rays analyzed with modified RECIST: PRs (*n* = 3), SD (*n* = 1) and NR (*n* = 6)	(45)
moDC	Allogeneic tumor cell lysate	9	MPM	Three biweekly vaccinations i.d. (1/3) and i.v. (2/3), followed by a boost at 3 and 6 months	CT scans analyzed with modified RECIST: PR (*n* = 2), SD (*n* = 7) Median PFS of 8.8 months and median OS not reached	(78)
moDC	Allogeneic tumor cell lysate	27	Prostate cancer	Twelve vaccinations s.c. at the axillary and inguinal areas; patients received 1 week of cyclophosphamide in metronomic setting prior to vaccinations	Increase of median PSADT from 5.67 (prior treatment) to 18.85 months (after treatment)	(48)
moDC	2 TAAs	15	NSCLC	Three vaccinations i.v. in 1-week intervals	Long-term follow-up until 2017: low dose group: no recurrence, progressive disease and death (*n* = 1 each); middle dose group: no recurrence (*n* = 3); high dose group: no recurrence (*n* = 7), progressive disease and death (*n* = 1)	(49)
moDC	3 TAAs	156	Hepatocellular carcinoma	Six injections s.c. near the inguinal lymph nodes over 14 weeks	Difference in RFS not statistically significant between treated and control groups; Significantly prolonged RFS in the treated non-radiofrequency ablation subgroup	(79)
moDC	TAA-mRNA	30	AML (in remission)	I.d. injections four times at 2-week intervals	Antileukemic effect (*n* = 13) with minimal residual disease (*n* = 9) or SD (*n* = 4); significantly higher OS and RFS compared to non-responders	(80)
moDC	4 HLA class I and 6 HLA class II peptides	53	Metastatic melanoma	Four vaccinations (at week 0, 2, 6, 10) followed after 2 months by 6 vaccination maintenance cycles for up to 2 years	No regression of all metastases according to WHO criteria but slow regression of individual metastases; 75% OS at 5 years in group of tumor-free patients; 19% of patients still alive after 12-year follow-up	(81)
moDC	6 HER2 MHC class II binding peptides	42	HER2^+^ breast cancer	Six weekly injections into the breast, into the groin LNs, or into both breast and in groin LNs	Higher pathologic complete response rate in ductal carcinoma *in situ* patients compared with invasive breast cancer patients (28.6% vs. 8.3%)	(74)
cDC2s	3 TAAs	14	Metastatic melanoma	Three i.n. injections once every 2 weeks; followed by 2 maintenance cycles of 3 biweekly vaccinations each with a 6-week interval	Long-term PFS of 12-35 months (*n* = 4) and median OS of 13.3 months	(47)
pDCs	3 TAAs	15	Metastatic melanoma	Three i.n. injections once every 2 weeks, followed by 2 maintenance cycles of 3 biweekly vaccinations with a 6-week interval	SD (*n* = 2), mixed response (*n* = 1); increased PFS (4.0 vs. 2.1 months) and OS (22.0 vs. 7.6 months) compared to 72 matched control (chemotherapy-treated) patients	(46)

*AML, acute myeloid leukemia; cDC2s, conventional DCs 2; CT, computed tomography; i.d, intra-dermal; i.n.,intra-nodal; i.v., intra-venously; moDCs, monocyte-derived DCs; MPM, malignant pleural mesothelioma; NR, no response; NSCLC, non-small cell lung carcinoma; PR, partial response; RECIST, Response Evaluation Criteria in Solid Tumors; OS, overall survival; pDCs, plasmacytoid DCs; PFS, progression-free survival; PSADT, prostate-specific antigen doubling time; RFS, recurrence-free survival; SD, stable disease; TAA, tumor-associated antigen; s.c. subcutaneous*.

A phase I clinical trial employed autologous tumor lysate-pulsed moDCs in ten patients with malignant mesothelioma after chemotherapy. Clinical responses were evaluated by modified RECIST. In addition, efficacy of DC vaccination was determined by increased cytotoxicity of isolated PBMCs against tumor cells and higher percentages of CD8^+^ T-cells expressing granzyme B, an indication for their capacity to lyse cells. After vaccination, four out of six patients showed increased cytotoxicity levels and granzyme B expressing CD8^+^ T-cells increased in nine patients (45). In another phase I clinical trial in nine patients with mesothelioma using allogeneic tumor cell lysate-pulsed moDCs, tumor-specific T-cells could be detected in the majority of patients in a skin biopsy after a positive DTH skin test. In addition, radiographic responses (two partial responses and seven patients with stable disease), progression free survival (8.8 months) and overall survival [(OS) not reached] of the patients were monitored and analyzed according to modified RECIST criteria (78). During one study in 27 prostate cancer patients with rising serum prostate-specific antigen [(PSA); indication for biochemical relapse of prostate cancer] levels, kinetics of PSA was monitored and used to determine the efficacy of the vaccination with moDC pulsed with allogeneic tumor cell lysate. The median PSA doubling time (PSADT), which determines clinical outcome, increased from 5.67 to 18.85 months. In addition, the frequency of PSA-specific T-cells increased after vaccination and tumor-specific IgG antibodies could be detected in nine patients. However, these immune response characteristics did not significantly correlate with PSADT (48). A recent phase I clinical trial in patients with NSCLC employed moDCs pulsed with two TAAs, silenced with SOCS1, and stimulated with flagellin. Upon vaccination, regulatory T-cells (Tregs) decreased, and three patients had increased levels of IL-6 and/or TNFα, whereas IL-2, IL-4, IL-10, and IFNγ were unaffected. These observed immune responses did not correlate with the clinical response (49). Another phase II trial in 156 patients with hepatocellular carcinoma (no residual tumor after standard treatment) investigated DC-based adjuvant immunotherapy using triple TAA-pulsed moDCs. While recurrence-free survival (RFS) and OS were not different between the immunotherapy and control (no treatment) groups, immunotherapy increased TAA-reactive T-cell responses and IFNγ levels, whereas levels of serum TGF-β decreased. Nevertheless, this did not correlate to RFS. Interestingly, when radiofrequency ablation (RFA) patients were excluded in *post-hoc* analyses, immunotherapy did prolong RFS of non-RFA patients (79).

In contrast, a phase II study in 30 patients with acute myeloid leukemia could correlate long-term OS with higher numbers of circulating TAA-specific CD8^+^ T-cells after therapy with moDCs electroporated with TAA-mRNA (80). Furthermore, a phase I/II clinical trial that studied the effectivity of DC vaccination in 62 patients with melanoma used moDCs loaded with 4 HLA class I peptides and 6 HLA class II peptides. DC vaccination increased the numbers of vaccines-specific IFNγ-producing T-cells, whereas numbers of Tregs and myeloid derived suppressor cells (MDSCs) were unaltered. Surprisingly, IFNγ-producing T-cells did not correlate with OS, whereas the intensity of allergic vaccine-injection site reactions significantly correlated with OS. Furthermore, a maximal eosinophilic blood count (>250 per 100 μl blood) significantly improved survival specifically in tumor bearing melanoma patients (81). Another study in 42 patients with HER2^+^ breast cancer, that used moDCs pulsed with six HER2 MHC class II binding peptides, could correlate pathologic complete response with the CD4^+^ Th1 immune response in the sentinel LN, but in peripheral blood (74).

**Figure 1:**
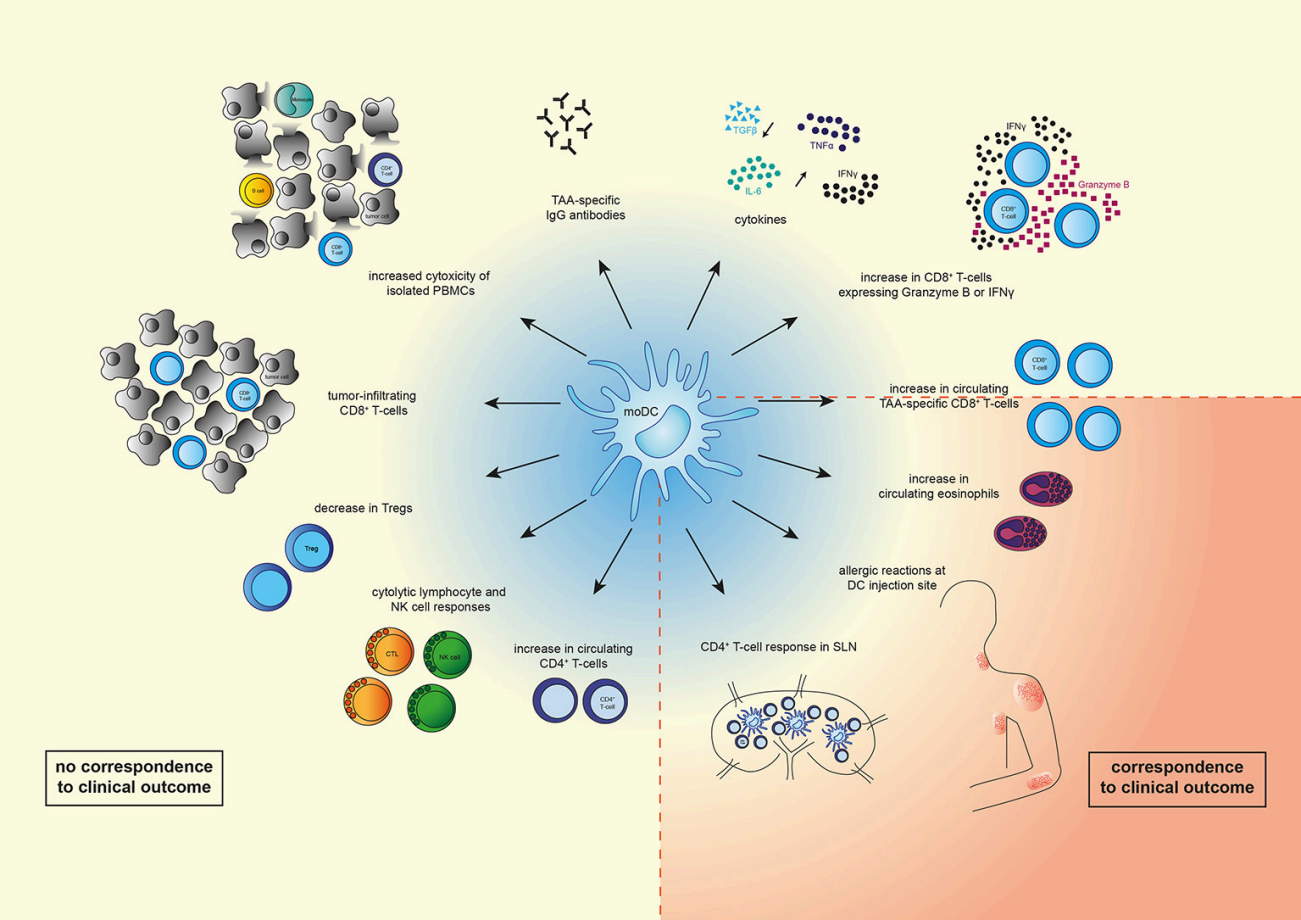
Overview of immunological changes observed upon moDC therapy. Vaccination with moDCs can lead to various immunological changes such as an increase in numbers of circulating immune cells (TAA-specific CD8^+^ T-cells, CD8^+^ T-cells expressing IFNγ or Granzyme B, CD4^+^ T-cells, eosinophils), or a decrease of other immune cells (Tregs). In addition, systemic cytolytic lymphocyte (CTL) or natural killer (NK) cell responses, as well as CD4^+^ T-cell responses in sentinel lymph nodes (LNs) were observed. Levels of TAA-specific IgG antibodies and cytokines (IL-6, IFNγ, TNFα) increased, whereas levels of TGFβ decreased. Vaccination with moDCs also resulted in tumor-infiltrating CD8^+^ T-cells, increased cytotoxicity of isolated PBMCs (monocytes, CD8^+^ and CD4^+^ T-cells, B-cells), and allergic reactions at the DC injection site. Of the shown changes, only increased circulating TAA-specific CD8^+^ T-cells, eosinophilic blood count, strength of allergic reactions at DC injection site, and a CD4^+^ T-cell response in sentinel LNs correspond to clinical outcome.

Overall, it seems that DC vaccination induced various immune responses, but most of the observed immunological responses do not reflect clinical responses (Figure 1). This could be due to the fact that most studies are phase I/II clinical trials in which safety and feasibility are the primary outcomes and not efficacy. Furthermore, this could be caused by the type and location of the immune response measured, as most studies focused on TAA-specific T-cells in peripheral blood. As DC vaccination initiates T-cell responses in the LNs and these TAA-specific T-cells exert their cytolytic function in the tumor, it would be more likely that immune responses in LNs or in the tumor predict OS better than immune responses measured in peripheral blood. This could be performed using a recently described method that can quantify tumor-specific CTLs in preclinical models at different sites (82).

Furthermore, it was shown in murine models that DC vaccines elicited cytotoxic and regulatory natural killer cell responses against tumors (83, 84). This stresses the necessity to investigate other cell subsets, besides T-cells, influenced by DC vaccines.

## Potential of Naturally Occurring DC Subsets for Use AS Vaccines

Despite the growing knowledge in DC immunobiology, the exact diversity and biology of T-cell responses generated by different DC vaccines is still poorly understood. The recent development of antibody-coated magnetic beads enables the isolation of natural occurring DC subsets directly from peripheral blood in considerate numbers. For example, more than 10 million pDCs or more than 27 million cDCs can be isolated from apheresis products (46, 47).

The first phase I/II clinical trials have been performed using naturally occurring DCs for DC therapy and have shown that this is safe and feasible (46, 85). One of the clinical trials that used naturally occurring cDC2s loaded with three TAAs in 14 melanoma patients showed that the presence of TAA-specific T-cells in peripheral blood and DTH tests correlated with progression-free survival in three patients (47). Another clinical study in 15 patients with metastatic melanoma used pDCs pulsed with three TAAs. Increased TAA-specific CD8^+^ T-cell frequencies were measured in the blood of seven of the fifteen patients. Clinical outcome (PFS and OS) of patients treated with TAA-loaded pDCs was increased as compared to 72 matched control patients treated with chemotherapy (46).

Unfortunately, it is unknown whether naturally occurring DCs outperform cultured moDCs as source for DC therapy in patients, as clinical trials comparing different DC subsets as a source for DC therapy have not been performed. However, in mice, efficacy of different DC subsets for DC-therapy was compared. Herein, they found that moDCs in the tumor are superior in antigen uptake and processing but failed to induce efficient T-cell proliferation. MoDCs in the tumor even seemed to have immunosuppressive properties, as they inhibited T-cell proliferation by increased iNOS expression (86), however this is likely dependent on environmental cues, as cultured moDCs are highly immunogenic. Tumoral cDC1s were superior in stimulating naïve and previously activated CD8^+^ T-cells, beneficial for tumors with abundant Tregs, whereas cDC2s purified from tumor were more efficient in CD4^+^ T-cell stimulation and differentiation into Th17 cells, which was effective for tumors with abundant M2-oriented tumor-promoting tumor-associated macrophages (TAMs) (86, 87). In another study of melanoma mouse models, cDC1s but not cDC2s were shown to transport intact TAAs to TdLNs and cross-present them to CD8^+^ T-cells (88). Whether these findings will be confirmed with *ex vivo* loading of natural occurring DCs remains to be determined, and is currently extensively studied.

## Implications for Future Design of Dendritic Cell Vaccines

The use of different natural occurring DC subsets for vaccination is promising and more studies directly comparing the various subsets are urgently needed. In addition, more research into the contribution of the DC subsets to the different aspects of anti-tumor immunity is required, as this can be beneficial for tumors with different composition of the TME.

It is known that different types of human solid tumors are infiltrated to various extents by different types of immune cells (89–91). The presence of these immune infiltrates even has prognostic value (92–94). Moreover, it might guide the choice of which DC type to employ for vaccination, as different DC subsets elicit differing T-cell responses against the tumor. Hence, identifying whether the immunosuppressive environment of the tumor consists Tregs or TAMs before treatment might help in choosing the right DC subset to induce the proper T-cell skewing.

Besides the direct (re)activation of tumor-specific T-cells, efforts are undertaken to combine DC vaccination with agents that can modulate the TME itself e.g., by immunotherapy, radiotherapy, or chemotherapy to act synergistically with DC vaccination, which can improve immunogenicity, T-cell infiltration, T-cell exhaustion, and overcome the immunosuppressive environment of the tumor (82, 95–98).

## Conclusion Remarks

Although DC vaccination has been optimized in recent years, a great potential for improvement still remains. More (pre)clinical studies investigating the working mechanisms underlying DC vaccine efficacy are required. Therein, a major focus should be laid on different DC (and other myeloid) subpopulations and their specialized contribution to antitumor immunity, as it is likely that different cancer types might need different DC therapeutic strategies.

## Author Contributions

All authors listed have made a substantial, direct and intellectual contribution to the work, and approved it for publication.

### Conflict of Interest Statement

JA: speakers fee and consultancy Eli-Lilly, Boehringer Ingelheim, MSD, BMS, Astra Zeneca, Amphera, Roche; Stock owner Amphera b.v. The remaining authors declare that the research was conducted in the absence of any commercial or financial relationships that could be construed as a potential conflict of interest.
